# Chemical Composition, Market Survey, and Safety Assessment of Blue Lotus (*Nymphaea caerulea* Savigny) Extracts

**DOI:** 10.3390/molecules28207014

**Published:** 2023-10-10

**Authors:** Noura S. Dosoky, Sara A. Shah, Joseph T. Dawson, Sushant Sharma Banjara, Ambika Poudel, Cécile Bascoul, Prabodh Satyal

**Affiliations:** 1 Essential Oil Science, dōTERRA International, Pleasant Grove, UT 84062, USA; ndosoky@doterra.com (N.S.D.); ssharmabanja@doterra.com (S.S.B.); apoudel@doterra.com (A.P.); 2 Product Safety, dōTERRA International, Pleasant Grove, UT 84062, USA; sshah@doterra.com (S.A.S.); jdawson@doterra.com (J.T.D.); cbascoul@doterra.com (C.B.)

**Keywords:** blue lotus, water lily, *Nymphaea caerulea*, aquatic plants

## Abstract

Blue lotus, also known as *Nymphaea caerulea* (Nymphaeaceae), is a water lily found globally in lakes and rivers. With its long history of use in Egyptian culture, blue lotus has been associated with spiritual rituals and health benefits. Nowadays, blue lotus is still consumed as a tea or tincture to induce relaxation and heightened spiritual awareness. In this study, six authentic *N. caerulea* extracts from trusted sources and eleven commercial products were analyzed using gas chromatography−mass spectrometry (GC-MS). Authentic blue lotus extracts were produced in industrial settings. Overall, the extracts were a mixture of aliphatic hydrocarbons, aromatic alcohols, fatty acids, phenyl derivatives, diterpenoids, phytosterols, and stigmastanes. Apomorphine and nuciferine, which are responsible for psychoactive effects of the blue lotus flower, were virtually absent from the authentic blue lotus extract. Although blue lotus has a long history of use, the safety data on the plant and its extracts is limited; however, together with the analytical data, the available information does not indicate major safety concerns for the topical application of authentic blue lotus flower concrete or absolute when diluted as a fragrance ingredient.

## 1. Introduction

Blue lotus, water lily, and Egyptian lotus are common names of *Nymphaea caerulea* Savigny (Nymphaeaceae). It is an aquatic perennial plant that grows globally along rivers and lakes at altitudes ranging from sea level to 2700 m asl [1]. *N. caerulea* is a recognized synonym of *N. nouchali* var. *caerulea* (Sav.) Verdc. [2]. The plant is characterized by its floating round or oval flat leaves (up to 40 cm in diameter) that arise from a perennial spongy rhizome submerged in the mud of pond habitats. The leaves stay afloat because of their top surface, which is covered with a smooth, waxy cuticle and slightly rolled-up margins [1]. The flowers of *N. caerulea* are its characteristic feature. Blooming in September until February, the flowers are presented in a star-like pattern when fully open, measuring about 15–20 cm in diameter. The flowers close in the afternoon after opening in mid-morning and can be found in a range of colors, such as blue, white, and pink, with blue being the most common.

Blue lotus has long-standing historical and cultural significance. Drawings and paintings of the blue lotus flower were reported on Egyptian papyri and tombs from the 14th century B.C., indicating its use in shamanistic rituals and health-related practices [3,4]. *Nymphaea* species were revered as the epitome of holiness and beauty in ancient Greece and Rome. Based on its regional distribution, the plant is classified into tropical and hardy water lilies [5]. Blue lotus is a popular ornamental plant used in landscaping and is used in water purification. The flowers, stems, and roots are used for health-related purposes [6,7]. In traditional medicine, *N. caerulea* is reputed to have calming and soothing effects. In Ayurvedic medicine, it is used for a variety of health-related issues [8]. The family Nymphaeaceae has been studied extensively in the field of pharmacognosy due to their ability to produce aminogenic secondary metabolites. These metabolites have been found to have a range of pharmacological activities including analgesic, anti-inflammatory, and antimicrobial properties. *N. caerulea* is a rich source of different secondary metabolites such as anthocyanins, anthraquinones, fatty acids, flavonoids, leuecoanthocyanins, phenols, coumarins, tannins, and triterpenoids [9,10,11,12]. The leaf and flower extracts are excellent sources of phytoconstituents when compared with the rhizome and root [11]. The flavonoid composition has been reported to determine the flower color. Cultivars with amaranth flowers contain delphinidin 3-galactoside, blue flowers contain delphinidin 3-O-galactoside, red flowers contain derivatives of delphinidin and cyanidin, while white and yellow flowers lack anthocyanins [13]. Because of its high content of polyphenols, blue lotus is recognized as a natural source of antioxidants that can delay food spoilage, slow down the aging process, support healthy cell growth, and promote cardiovascular health [12,14]. Kaempferol, quercetin, quercitin, chalcone, and gallic acid have been identified from the plant [12,15,16]. According to Agnihotri et al. [7], the ethyl acetate fraction of *N. caerulea* flowers and nine isolated compounds can be used as a natural solution for oxidative stress. The blue lotus flower has been chiefly utilized in relation to relaxation and sleep in modern times. At high doses, some users might experience hallucinations and euphoria [4]. In a case series, five active-duty patients presented to the emergency department with altered mental status following the use of blue lotus products, four after vaping and one after making an infused beverage [4]. Although the case series did not include confirmatory analytical data, the effects were attributed to two compounds, apomorphine and nuciferine, which were previously found to be present in these types of products [17]. Interestingly, these two compounds have been studied and used as therapeutic agents using oral doses in the range of 15–150 mg/day [18,19]. Little is known about industrially produced blue lotus extracts. Currently, various blue lotus products are accessible online including dried leaves, teas, plant resins, flower extracts, oils, concentrated alkaloids, and electronic cigarette liquids [17]. These products are labeled as natural, but mostly have not been approved by the Federal Drug Administration (FDA) for human consumption. Therefore, we aimed to investigate the chemical composition of industrially produced floral extracts of *N. caerulea* and compare the composition to the commercial products available in the U.S. market. Moreover, we assessed the safety of *N. caerulea* extracts.

## 2. Results and Discussion

### 2.1. Authentic Blue Lotus Extracts

Authentic blue lotus extracts were produced in industrial settings. The average yields were 0.18% and 0.09% for the concrete and absolute, respectively. The aroma of *N. caerulea* extracts can be described as floral, fruity, sweet, fig-like, leathery, and slightly herbaceous. The volatile fraction ranged from 38.7–65.1% of the total extract. Table 1 summarizes the chemical compositions of authentic *N. caerulea* extracts. Overall, the extracts were a mixture of aliphatic hydrocarbons, aromatic alcohols, fatty acids, phenyl derivatives, diterpenoids, phytosterols, and stigmastanes. The chemical makeup of the flower is quite complex. Fossen and coworkers identified seven flavonoids and five anthocyanins, including three acylated anthocyanins from the methanolic extract of the flower [20,21]. Agnihotri and colleagues isolated and identified several compounds from the flower ethanolic extract with a considerable antioxidant activity [7]. In a study using headspace solid-phase microextraction (HS-SPME) followed by GC-MS analysis, the vapor phase of a trapped *N. caerulea* live flower contained benzyl acetate (10.4%), pentadecane (15.5%), 6,9-heptadecadiene (40.1%), and 8-heptadecene (15.3%) as the main components [22]. When the stamens, pistils, and petals were compared, it was reported that the majority of volatiles were produced by the stamens, with alkanes, alkenes, aldehydes, and ketones being the most abundant [23].

### 2.2. Alkaloids

Alkaloids are mainly found in lotus leaves [24,25]. Nuciferine is insoluble in water and soluble in acidic aqueous solutions and organic solvents such as chloroform, ethanol, and methanol [24]. While acid-ethanol extraction was traditionally used to extract nuciferine, ultrasound-assisted acid-ethanol extraction seemed to improve the results [24]. In the current study, blue lotus concretes and absolutes were free of apomorphine and contained negligible traces of nuciferine (10–72 ppb). This finding indicates that the extraction conditions to produce concrete and absolute using hexane followed by ethanol were not optimal for their extraction.

### 2.3. Commercial Products

Eleven commercially available blue lotus products were purchased online. Interestingly, the aroma varied greatly between the commercial products and none of these products resembled the original aroma. More than 150 compounds were identified from the obtained products (Table 2). All of the tested samples contained synthetic fragrance components. Unlike the authentic samples, terpenes were among the identified compounds. C1, C2, and C7 showed signs of a *Citrus* oil, *Lavandula* oil, and *Geranium* oil addition, respectively. It was hard to recognize which oil was added to C8-C11. Furthermore, there is evidence that herculyn D was used as a fragrance fixative in C4, C8, and C9, as indicated by the presence of abietic acid derivatives.

### 2.4. Safety Assessment

*N. caerulea* is not GRAS classified, and no published safety data were found on the plant or the extracts as a whole. However, the plant has a long history of use. The safety data for all constituents present at 1% and above are presented in Table 3. The three main constituents detected in the concrete were 6,9-heptadecadiene (11.95 ± 1.65%), n-tricosane (8.03 ± 0.18%), and benzyl alcohol (7.58 ± 0.85%). The three main constituents identified in the absolute were 6,9-heptadecadiene (11.05 ± 1.53%), benzyl alcohol (10.46 ± 1.42%), and tetradecanol (5.32 ± 0.74%). Tsai et al. [22] studied the volatile compounds of *N. caerulea* (water lily) flowers using GC-MS and reported four main compounds: 6,9-heptadecadiene (40.1%), pentadecane (15.5%), 8-heptadecene (15.3%), and benzyl acetate (10.4%). This is different from our GC-FID results, except that the main compound, 6,9-heptadecadiene, was identified as the most abundant compound, although at a substantially lesser concentration in the flower extracts.

As these are absolute and concrete materials, they may contain an unknown and significant portion of nonvolatile compounds. As such, quantification through GC-FID may not be accurate and compounds present in the absolute and concrete may not be detected and therefore not evaluated as part of this assessment. Since the safety of unidentified compounds cannot be guaranteed, this presents an unknown safety risk.

Of the 28 compounds investigated (making up 85.43% of the concrete and 80.52% of the absolute), safety information was not found for 10 compounds (making up 27.29% of the concrete and 25.11% of the absolute) including 6.9-heptadecadiene, n-pentacosane, tetrapenol, 3-((8Z,11Z)-heptadeca-8,11-dien-1-yl)-5-methoxyphenol, 2-nonadecanone, heptacosane, benzyl linoleate, methyl cholesterol, benzyl linolenate, and benzyl hexadecanoate. We were able to gather safety information for the remaining 18 compounds (making up 58.69% of the concrete and 56.68% of the absolute) including n-tricosane, benzyl alcohol, tetradecanol, heneicasane, nonadecane, pentadecane, oleic acid, E-squalene, linoleic acid, γ-sitosterol, palmitic acid, phytol, E-β-farnesene, ethyl stearate, stigmasterol, hexadecyl acetate, ethyl linoleate, and ethyl palmitate. According to the data available from CIR, all of the assessed compounds, except two, were found to be at concentrations considered safe in accordance with current usage practices, as indicated in Table 3. Benzyl alcohol and tetradecanol are slightly above the maximum concentrations; however, when blue lotus extracts are used as part of a formulation, the concentration of these compounds will be reduced. For compounds with data on genotoxicity, there was no indication of genotoxicity risks. Very limited information was available on acute or chronic toxicity and phototoxicity or photoallergenicity. However, the data are not indicative of major safety risks.

## 3. Materials and Methods

### 3.1. Plant Material and Extraction

**Figure 1:**
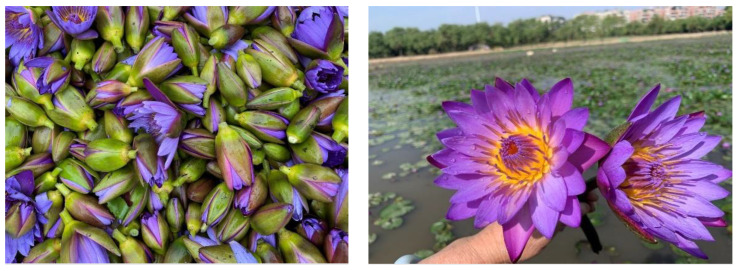
Fresh *Nymphaea caerulea* flowers harvested from the field.

Authentic blue lotus absolutes and concrete samples were prepared using industrial extraction methods. Cultivated blue lotus plants were collected from Hainan and Guangdong, China (Figure 1). The plant prefers high temperatures, humidity, and sunlight. Fresh flowers were shredded with a flower-cutting machine. About 1000 Kg of the shredded material was extracted twice with hexane (1: 2, *w*/*v*) in an enamel extraction tank with continuous stirring for 12 h. After soaking, the hexane was discharged and filtered with 120 mesh stainless steel mesh. The collected extracts were allowed to settle for 4 h, then filtered. The solvent was then recovered by heating with jacketed steam. The extract was concentrated under atmospheric pressure with a spherical concentrator until all of the hexane was evaporated. The concentrated extract is called concrete. To prepare the absolute, the blue lotus concrete was dewaxed with 95% ethanol (1:5, *w*/*v*) in a stainless-steel barrel, stirred carefully, and placed in the freezer for more than 12 h. The resulting extract was filtered and the floral was separated. The filtrate was concentrated under low pressure in a spherical concentrator until all of the solvent was evaporated. Samples of both the concrete and absolute were tested for solvent residue. Eleven commercially available blue lotus oil products were purchased online (Amazon and Etsy). The product labels of these samples contained the information listed in Table 4.

### 3.2. Gas Chromatography−Mass Spectrometry (GC–MS) Analysis

Authentic and commercial samples were analyzed using a gas chromatograph coupled to a mass spectrometer QP2010 Ultra (Shimadzu Scientific Instruments, Columbia, MD, USA) with electron impact (EI) mode with 70 eV, as previously described [55]. The components were identified by comparing the mass spectral fragmentation patterns (over 80% similarity match) and retention indices (RI) based on a series of homologous C8-C20 *n*-alkanes with those reported in databases (NIST database, and our in-house library) using the Lab Solutions GCMS post-run analysis software version 4.45 (Shimadzu Scientific Instruments, Columbia, MD, USA).

### 3.3. Gas Chromatography–Flame Ionization Detection (GC–FID) Analysis

Analysis of *E. purpurea* essential oil was carried out using a Shimadzu GC 2010 equipped with a flame ionization detector (Shimadzu Scientific Instruments, Columbia, MD, USA), as previously described [56], with a ZB-5 capillary column (Phenomenex, Torrance, CA, USA).

### 3.4. Detection and Quantification of Nuciferine and Apomorphine

LCMS-grade methanol, LCMS-grade water, and HPLC-formic acid were purchased from Sigma-Aldrich (St. Louis, MO, USA). Nuciferine and apomorphine were purchased from Cayman Chemical (Ann Arbor, MI, USA). Stock solutions of each standard at a concentration of 10 ppm were prepared by diluting the powder in methanol. Nuciferine and apomorphine were quantified using a NEXERA UPLC system (Shimadzu Corp., Kyoto, Japan) equipped with a mass spectrometer (Triple quadrupole, LCMS8060, Shimadzu, Kyoto, Japan) as previously described [18,25]. The detection was completed in multiple reaction monitoring mode (MRM) (Table 5). Samples were run in triplicate with external standards in between and the injection volume was 1 μL. The acquired chromatographic results were processed in LabSolutions Insight software version 3.2 (Shimadzu). For each compound, calibration curves (0.005–0.1 ppm) were created by linking the peak area and the concentration.

### 3.5. Safety Assessment

The safety assessment of blue lotus extracts was conducted by applying standard toxicology and risk assessment methods using the analytical results (Table 2), published safety data on the raw material as a whole plant, plant extract, and the constituents identified in the extracts. The information considered for the safety assessment included the historical use of the plant and extracts, safety and toxicology data on the plant and extracts, and safety and toxicology data of all constituents present at 1% and above. This safety assessment is based solely on the available literature. The documents collected and reviewed included scientific articles from books and scientific journals on botany and the safety of natural complex substances, fragrances, and flavors. Studies using different degrees of evidence from in vitro methods, pre-clinical models, clinical trials, and case reports were used as evidence of the safety or toxicity of the raw material as a whole. The sources of information used to evaluate the safety of individual constituents included the RIFM (Research Institute for Fragrance Materials, Inc.) Fragrance and Flavor Database, CIR (Cosmetic Ingredient Review) assessments, and ECHA (European Chemical Agency) REACH (Registration, Evaluation, Authorization, and Restriction of Chemicals) registrations. The main endpoints of interest included genotoxicity, developmental and reproductive toxicity, skin irritation and sensitization, photoirritation and photoallergenicity, as well as acute and chronic toxicity for oral, dermal, and inhalation routes of exposure.

## 4. Conclusions

In this study, we analyzed the chemical composition of six authentic blue lotus extracts and eleven commercial products. The extracts were a mixture of aliphatic hydrocarbons, aromatic alcohols, fatty acids, phenyl derivatives, diterpenoids, phytosterols, and stigmastanes. The main constituents in the authentic concrete were 6,9-heptadecadiene (11.95 ± 1.65%), *n*-tricosane (8.03 ± 0.18%), and benzyl alcohol (7.58 ± 0.85%), while the main constituents of the authentic absolute were 6,9-heptadecadiene (11.05 ± 1.53%), benzyl alcohol (10.46 ± 1.42%), and tetradecanol (5.32 ± 0.74%). Surprisingly, none of the investigated commercial products resembled authentic extracts in aroma or composition. Nuciferine and apomorphine were found in traces or were absent, respectively, from the studied authentic extracts, suggesting that the risk of psychoactive effects associated with these compounds would be virtually absent for a small dose of either of these extracts applied topically. Other than the psychoactive effects associated with nuciferine and apomorphine, the available safety data from the literature are limited and do not show major safety concerns for the authentic extracts. Surprisingly, none of the investigated commercial products resembled authentic extracts in aroma or composition.

## Figures and Tables

**Table 1:** Chemical composition of authentic blue lotus extracts.

RI_exp_^a^	RT	Compound Name	Concrete (Area %)					Absolute (Area %)				
			1	2	3	Avg	SD	1	2	3	Avg	SD
1038	13.253	Benzyl alcohol	7.94	8.20	6.61	7.58	0.85	12.09	9.74	9.53	10.46	1.42
1166	19.126	Benzyl acetate	0.21	0.10	0.17	0.16	0.05	0.28	0.20	0.13	0.20	0.07
1284	24.686	*p*-Anisyl alcohol	0.59	0.41	0.15	0.38	0.23	1.15	0.79	0.80	0.91	0.20
1435	31.304	*(E)*-α-Bergamotene	0.28	0.28	0.36	0.30	0.05	0.27	0.37	0.38	0.34	0.06
1454	32.119	*(E)*-β-Farnesene	1.43	1.27	1.48	1.39	0.11	1.44	1.59	1.87	1.63	0.22
1500	34.135	Pentadecane	4.24	4.16	5.53	4.64	0.77	3.41	4.40	5.01	4.27	0.81
1504	34.275	*(E,E)*-α-Farnesene	0.73	0.63	0.78	0.71	0.08	0.73	0.86	1.04	0.88	0.16
1525	35.084	β-Sesquiphellandrene	0.64	0.63	0.83	0.70	0.11	0.59	0.80	0.82	0.74	0.13
1670	40.752	6,9-Heptadecadiene	11.10	10.89	13.85	11.95	1.65	9.80	11.96	12.75	11.50	1.53
1674	40.901	*(Z,Z,Z)*-1,8,11,14-Heptadecatetraene	0.48	0.46	0.61	0.52	0.08	0.45	0.55	0.62	0.54	0.08
1678	41.058	Tetradecanol	5.51	5.46	7.01	5.99	0.88	4.56	5.37	6.04	5.32	0.74
1697	41.823	2-Pentadecanone	0.21	0.15	0.21	0.19	0.03	0.31	0.31	0.31	0.31	0.00
1700	41.932	Heptadecane	0.50	0.87	1.15	0.84	0.33	0.71	0.78	0.84	0.78	0.07
1837	46.789	Neophytadiene	0.52	0.42	0.48	0.47	0.05	0.23	0.25	0.26	0.25	0.01
1900	48.999	Nonadecane	4.78	4.45	4.72	4.65	0.18	4.51	3.10	2.90	3.50	0.88
1908	49.234	*(E,E)*-7,11,15-Trimethyl-3-methylene-hexadeca-1,6,10,14-tetraene	0.34	0.26	0.28	0.29	0.04	0.38	0.32	0.33	0.34	0.03
1959	50.907	Palmitic acid	2.22	1.81	1.91	1.98	0.22	3.11	2.57	2.28	2.65	0.42
1993	52.041	Ethyl Palmitate	0.44	0.69	0.52	0.55	0.13	1.31	1.16	1.33	1.27	0.09
2008	52.512	Hexadecyl acetate	0.87	0.94	1.27	1.03	0.21	0.83	1.39	1.03	1.08	0.28
2101	55.438	Heneicosane	4.52	4.47	5.55	4.85	0.61	1.88	2.20	2.57	2.22	0.35
2104	55.544	2-Nonadecanone	1.50	1.63	1.78	1.64	0.14	0.17	0.10	0.20	0.16	0.05
2108	55.649	Phytol	1.92	1.46	1.23	1.53	0.35	2.74	2.09	2.21	2.35	0.35
2129	56.277	Linoleic acid	3.26	2.61	2.57	2.81	0.38	4.77	3.50	3.52	3.93	0.73
2135	56.454	Oleic Acid	4.29	4.26	4.10	4.22	0.10	6.12	4.42	4.38	4.97	1.00
2159	57.189	Ethyl Stearate	1.16	1.42	1.01	1.20	0.21	2.75	1.09	1.45	1.76	0.87
2165	57.363	Ethyl linoleate	0.85	1.15	0.85	0.95	0.17	2.34	2.14	1.51	2.00	0.43
2180	57.832	Tetrapenol	3.12	3.12	2.69	2.98	0.25	4.42	3.30	3.32	3.68	0.64
2287	60.929	*(E,E,E)*-2,6,10,14-Hexadecatetraen-1-ol 3,7,11,15-tetramethyl acetate	0.49	0.47	0.41	0.46	0.04	0.56	0.48	0.54	0.53	0.04
2301	61.34	*n*-Tricosane	7.84	8.06	8.19	8.03	0.18	1.39	2.27	2.62	2.09	0.63
2400	64.105	*n*-Tetracosane	0.23	0.24	0.22	0.23	0.01					
2501	66.78	*n*-Pentacosane	3.04	3.19	2.91	3.05	0.14	0.23	0.56	0.58	0.46	0.19
2577	68.726	Benzyl hexadecanoate	0.81	0.73	0.81	0.78	0.04	1.06	1.49	1.59	1.38	0.28
2600	69.344	*n*-Hexacosane	0.12	0.14	0.19	0.15	0.04					
2683	71.394	*n*- Heptacosane	1.54	1.69	1.51	1.58	0.10	0.51	0.71	0.48	0.57	0.12
2749	73.056	Benzyl linoleate	1.36	1.23	1.22	1.27	0.08	1.73	2.27		2.00	0.38
2757	73.256	Benzyl linolenate	0.83	0.71	0.82	0.79	0.06	1.17	1.58	1.73	1.49	0.29
2779	73.833	Benzyl stearate	0.20	0.13	0.18	0.17	0.04	0.22	0.24	0.28	0.25	0.03
2790	74.114	Octacosane	0.12	0.09	0.14	0.12	0.03					
2803	74.447	*(E)*-Squalene	3.67	4.09	3.78	3.85	0.22	2.30	2.23	2.16	2.23	0.07
2880	76.512	3-((8*Z*,11*Z*)-Heptadeca-8,11-dien-1-yl)-5-methoxyphenol	2.53	2.97	1.09	2.20	0.98	2.79	2.48	2.60	2.62	0.16
3023	80.457	β-Sitosterol acetate	0.25	0.35	0.14	0.25	0.10	0.20	0.13		0.17	0.05
3055	81.361	Vitamin E	0.22	0.21	0.21	0.21	0.00	0.26	1.02	1.12	0.80	0.47
3123	83.327	Methyl cholesterol	1.047	1.11	0.99	1.05	0.06	1.18	1.29	1.28	1.25	0.06
3142	83.885	Stigmasterol	0.97	1.12	1.11	1.07	0.08	1.20	1.28	1.28	1.25	0.05
3184	85.156	γ-Sitosterol	2.83	2.13	2.14	2.37	0.40	3.34	3.90	3.87	3.70	0.32
		Total identified %	91.73	90.85	93.76			89.47	87.28	87.52		

RI_exp_ = experimental retention index, RT = retention time, ^a^ retention index determined with respect to a homologous series of *n*-alkanes on a ZB-5ms column.

**Table 2:** Chemical composition of commercial (C) samples.

RI	Compounds	C1	C2	C3	C4	C5	C6	C7	C8	C9	C10	C11
778	Isobutyl acetate	0.12										
931	α-Pinene	0.06	0.09	0.04			0.04	tr			0.07	0.07
948	Camphene						0.01			0.03		0.01
962	Benzaldehyde	0.02	0.17				0.01					
967	Glycerin					58.33						
971	Sabinene	0.06					0.01				0.02	0.03
978	β-Pinene	0.37	0.18	0.14			0.03				0.36	0.38
983	3-Octanone						0.05					
988	Myrcene	0.05					0.05				0.04	0.05
991	2,6-Dimethyl-2-heptanol			0.05			0.01	0.01			0.04	0.05
997	Diethyl diglycol				0.24		0.84	0.78	0.42	0.33		
1010	Hexyl acetate						0.04					
1013	1,4-Cineole			0.06							0.04	0.04
1019	*p*-Methyl anisole		0.15									
1023	*p*-Cymene	0.09		6.92							3.00	2.17
1024	Dipropylene glycol 1										0.69	2.61
1026	Dipropylene glycol 2			6.03							0.90	6.04
1027	Limonene	3.08	0.80	3.48	0.03		0.29		0.04	0.06	3.75	4.21
1031	1,8-Cineole			0.05	0.21		0.07		0.28	0.28	0.09	
1033	(*Z*)-β-Ocimene		0.17				0.37					
1034	Benzyl alcohol							0.01				
1043	Dipropylene glycol 3			0.64							1.91	4.66
1044	(*E*)-β-Ocimene						0.01					
1045	(*E*)-β Ocimene						0.01					
1046	Dipropylene glycol 4			4.67							6.04	1.11
1049	Dipropylene glycol 5			1.06							0.73	
1056	Dipropylene glycol 6										4.66	
1057	γ-Terpinene	0.22	0.11	0.23							0.38	0.55
1069	(*Z*)-Linalool oxide (furanoid)		0.29					0.01				
1069	Dihydro myrcenol	3.22		3.40							3.62	3.42
1080	Dipropylene glycol 7										0.66	
1085	Terpinolene			0.05			0.02				0.02	0.10
1086	(*E*)-Linalool oxide (furanoid)		0.27				0.01					
1090	3-(*Z*)-Hexenyl methyl carbonate			0.04							0.03	0.04
1094	Methyl benzoate		0.31									
1098	Linalool	6.85	12.38	12.33			4.73	2.22	2.64	2.64	12.75	11.69
1114	Phenyl ethyl alcohol		0.31		1.87		7.01	7.74	4.11	3.79		
1118	3-Octanol acetate						0.37					
1127	*allo*-Ocimene						0.01					
1127	(*E*)-Rose oxide							0.02				
1134	Dihydro linalool	0.07	0.08				0.04	0.02				
1149	Camphor				2.47		0.02		0.29	0.29		
1151	Citronellal							0.04				
1157	Menthone							0.30				
1161	Benzyl acetate		0.55		1.23		2.26	2.07	1.16	1.23		
1164	Borneol											
1166	Isomenthone							0.15				
1170	Isononyl acetate			0.19							0.19	0.18
1174	2-Phenyl ethyl formate							0.07				
1178	(*Z*)-Pinocamphone						0.02					
1181	Terpinen-4-ol		0.18		0.07		0.37		0.12	0.12		
1192	Methyl salicylate		0.31									
1194	Dihydro citronellol							0.01				
1195	α-Terpineol			0.08	0.09		0.16	0.01	0.15	0.15	0.08	0.08
1198	Florosa				0.44		0.32	0.77	0.48	0.41		
1215	(*E*)-Rozanol				1.52		1.29	3.93	2.20	2.03		
1225	Citronellol	0.69	1.39		3.12		8.55	11.77	4.68	4.18		
1225	Nerol						0.81	1.71				
1226	Sabinene hydrate acetate										0.02	
1239	Neral							0.02				
1247	Linalyl acetate	7.78	2.92	5.14	1.70		3.53		2.43	2.36	5.19	5.10
1248	Geraniol				0.40		1.72	3.23	2.15	1.37		
1267	Dihydro linalyl acetate	0.10		0.05			0.03				0.07	0.07
1267	Geranial			0.03				0.03				
1269	1-Isoprpyl-3-tert-butylbenzene						0.04	0.04				
1272	Citronellyl formate							0.79				
1275	Neryl formate							0.04				
1279	Lavandulyl acetate		0.14									
1286	Hydroxy citronellal		7.43	0.19	2.01		3.59	4.07	2.39	2.38	0.18	0.20
1288	(*Z*)-2-tert-butyl cyclohexanol acetate	1.01										
1292	Indole				0.07		0.09	0.09	0.08	0.09		
1297	Geranyl formate							0.16				
1330	2-Propanol, 1,1’-[(1-methyl-1,2-ethanediyl)bis	2.51										
1345	α-Terpinyl acetate	0.06		0.03							0.03	0.03
1347	Citronellyl acetate							0.22				
1354	Neryl acetate	0.16	0.21		0.05		0.20		0.07	0.06		0.02
1374	Geranyl acetate	0.29	0.52	0.34	0.11		0.12	0.21	0.16	0.15	0.34	0.36
1377	α-Copaene		0.2									
1380	α-α-α-2-Trimethyl benzeneacetic						0.02	0.03				
1388	(*E*)-α-Damascone	0.17		0.03							0.02	0.03
1395	Vanillin		0.63									
1408	β-Maaliene		0.17									
1410	Calone	0.15		0.27							0.27	0.32
1421	β-Caryophyllene		1.10		0.08		0.42	0.01	0.11	0.11		
1423	Allyl cyclohexyl propanoate			0.06							0.05	0.07
1438	Coumarin	0.22										
1442	Dihydro curcumene	0.23	1.47									
1443	(*E*)-Cinnamyl acetate		0.29									
1446	(*E*)-Isoeugenol		0.21									
1447	1-(4-tert-Butylphenyl)propan-2-one	0.16		0.05			0.24	0.28	0.10	0.10		
1449	(*E*)-β-farnesene		0.25									
1458	α-humulene		0.09									
1460	Cyclamanal				0.76		1.86	1.73	0.94	0.94		
1465	γ-Decalactone	0.55	0.93									
1472	Isomethyl-α-(*Z*)-ionone	1.15										
1478	(*E*)-β-Ionone			0.29							0.27	0.31
1480	Sandal mysore core				1.81							
1501	Butylated hydroxy toluene			0.22							0.21	0.26
1502	α-Bulnesene		0.08									
1513	6-Methyl α-ionone	0.34										
1527	(*Z*)-Nerolidol		0.08									
1529	Lilial	5.65		13.68	9.34		16.93	15.43	9.69	10.13	13.79	14.04
1538	(*E*)-α-Bisabolene		0.07									
1549	Raspberry ketone	0.19										
1553	Geranyl butyrate							0.01				
1560	(*E*)-Nerolidol		0.15	0.31			0.29				0.28	0.30
1565	Tropional	0.88										
1569	Methyl-β-ionone	0.13										
1570	γ-Undecalactone			0.18							0.19	0.19
1585	Caryophyllene oxide							0.28				
1625	γ-Eudesmol							0.07				
1626	Cedryl methyl ether			0.25							0.25	0.31
1649	(*Z*)-Methyl dihydro jasmonate	2.29	10.26	21.79	6.75		12.73	12.02	7.34	7.30	21.37	20.66
1656	(7-α-Isopropenyl-4,5-dimethyl octahydroinden-4-yl)methanol											2.54
1659	Lyral	1.71	9.70		6.67		9.29	9.38	5.46	5.41		
1669	3-(*Z*)-Hexenyl salicylate	0.47	5.83									
1669	*Iso*-(*E*)-γ-Super			2.17							2.20	0.58
1675	(*E*)-Methyl dihydro jasmonate	0.27	1.41	2.34	0.80		2.16	0.05	0.86	0.84	2.49	2.65
1678	Salicylic acid hexyl ester		1.94					9.74				
1693	*Iso*-(*E*)-α-Super			0.44							0.53	
1696	2-(*Z*)-6-(*Z*)-Farnesol		0.35									
1729	2-Methoxy ethoxy cyclododecane				0.86		1.73	1.74	0.97	0.98		
1746	2-Hexyl-(*E*)-cinnamaldehyde	11.84		2.56	4.34		8.29	0.47	4.86	4.85	2.62	2.95
1756	Cosmone isomer II			0.04	0.07						0.04	
1768	Ambroxide						0.26	0.21	0.18	0.18		
1768	Benzyl benzoate		0.56									
1769	Methyl cedryl ketone	2.41										
1770	2-Hexyl-(*Z*)-cinnamaldehyde			0.16			0.45		0.34	0.32	0.11	0.16
1802	(*Z, E*)-Farnesyl acetate		0.08									
1827	(*E,E*)-Farnesyl acetate											
1844	Acetyl methyl tetralin	10.15		5.03							4.72	5.96
1870	Galaxolide 1	0.24		0.16							0.15	0.21
1871	Benzyl salicylate		0.39									
1875	Galaxolide 2	0.18		0.14							0.13	0.19
1892	Galaxolide 3	0.31		0.2							0.18	0.25
1903	Galaxolide 4	0.31		0.19							0.18	0.23
2011	Ethylene brassylate		1.10	1.89							1.83	2.02
2057	Ricenalidic acid lactone					8.40						
2098	Benzyl cinnamate		0.03									
2234	Methyl Pimarate				0.09				0.13	0.11		
2249	Methyl pimar-8(14)-en-18-oate				1.72				2.02	6.08		
2290	Methyl pimaran-18-oate				2.24				2.89	2.96		
2300	Methyl-8-piramen-18-oate isomer I				2.64							
2311	trans-3-Phenylpropyl cinnamate		0.54									
2315	Methyl 13-abieten-18-oate				2.98				3.03	2.99		
2324	Methyl-8-piramen-18-oate isomer II				23.82				5.84			
2330	Methyl 7-isopimaren-18-oate				0.29				0.20	0.20		
2338	Methyl dehydroabietate				6.47				6.17			
2360	Methyl abiet-7-en-18-oate				0.56				0.58	0.62		
2387	Methyl abietate				1.82				1.87	2.07		
2420	Cinnamyl cinnamate		0.95									
2435	Methyl neoabietate				0.18				0.23	0.25		
2695	Verdantiol isomer II			0.07	0.05						0.05	0.09
2927	Tricaprylin Triglyceride				1.68							
3084	β-Sitosterol acetate					7.54						
3116	Caprin Biscaprylin Triglyceride				3.19							
3301	Caprylin Biscaprin Triglyceride				1.82							
3325	β-Amyrone		0.17									
3376	Lupenone		2.85									
3486	Tricaprin Triglyceride				0.40							
	Total	66.81	70.84	97.76	97.06	74.27	91.82	91.99	77.66	68.39	97.83	97.59
	**Unidentified**	**33.2**	**28.8**	**2.19**	**2.75**	**25.69**	**8.18**	**7.88**	**22.24**	**31.59**	**2.13**	**2.38**

**Table 3:** Toxicological reference values from CIR, RIFM, and ECHA for compounds ≥1% identified in authentic blue lotus extracts.

Compound Name	CAS Number	Average Concentration (%)		CIR	RIFM					ECHA					Ref.
				Max Use Concentration	Genotoxicity	Phototoxicity	NOAEL (mg/kg/day)		NESIL (ug/cm^2^)	LD50			Repeated Dose ^±^		
		Concrete	Absolute				Repeated Dose	Developmental & Reproductive		Oral (mg/kg)	Dermal (mg/kg)	Inhalation (mg/L)	Oral NOAEL (mg/kg/d)	Inhalation NOAEC (mg/m^3^)	
6,9-Heptadecadiene	-	11.95%	11.50%	-	-	-	-	-	-	-	-	-	-	-	N.A.
*n*-Tricosane	638-67-5	8.03%	2.09%	-	NIG	-	-	-	-	-	-	-	-	-	[26]
Benzyl alcohol	100-51-6	7.58%	10.46%	≤10%	NG	NPT/A	100 ^†^	500	5900	1620	>2000	>4.2	400 *	1072 ^‡^	[27,28,29]
Tetradecanol	112-72-1	5.99%	5.32%	<5%	NIG	-	-	-	-	>2000	8000	>1.5	3548 ^‡^	1000 ^†^	[30,31,32]
Heneicosane	629-94-7	4.85%	2.22%	-	NIG	-	-	-	-	-	-	-	-	-	[33]
Nonadecane	629-92-5	4.65%	3.50%	-	NIG	-	-	-	-	-	-	-	-	-	[34]
Pentadecane	629-62-9	4.64%	4.27%	-	NIG	-	-	-	-	>5000 ^#^	>2000 ^#^	>6.0 ^#^	≥500 ^#,†^	≥6000 ^#,†^	[35,36]
Oleic acid	112-80-1	4.22%	4.97%	≤20.9%	NIG	-	-	-	-	-	-	-	-	-	[37,38]
(*E*)-Squalene	111-02-4	3.85%	2.23%	≤10%	-	-	-	-	-	>5000	-	13,800	>600 ^‡^	-	[39,40]
*n*-Pentacosane	629-99-2	3.05%	0.46%	-	-	-	-	-	-	-	-	-	-	-	N.A.
Tetrapenol	24034-73-9	2.98%	3.68%	-	-	-	-	-	-	-	-	-	-	-	N.A.
Linoleic acid	60-33-3	2.81%	3.93%	≤21.8%	NIG	-	-	-	-	-	-	-	-	-	[37,41]
γ-Sitosterol	83-47-6	2.37%	3.70%	≤10%	-	-	-	-	-	-	-	-	-	-	[42]
3-((8*Z*,11*Z*)-Heptadeca-8,11-dien-1-yl)-5-methoxyphenol	-	2.20%	2.62%	-	-	-	-	-	-	-	-	-	-	-	N.A.
Palmitic acid	57-10-3	1.98%	2.65%	≤21%	NG	NPT/A	-	-	-	>5000	>2000 ^#^	>0.15 ^#^	1000–5000 ^#,†^	-	[37,43,44]
2-Nonadecanone	629-66-3	1.64%	0.16%	-	-	-	-	-	-	-	-	-	-	-	N.A.
Heptacosane	593-49-7	1.58%	0.57%	-	-	-	-	-	-	-	-	-	-	-	N.A.
Phytol	150-86-7	1.53%	2.35%	-	NG ^#^	NPT/A	333 ^#,†^	-	2700 ^#^	>10,000	>4000	-	100	-	[45]
(*E*)-β-Farnesene	18794-84-8	1.39%	1.63%	-	NG ^#^	NPT/A ^#^	-	-	3700 ^#^	>5000	>5000	>2.06	≥1000 ^†^	-	[46,47]
Benzyl linoleate	47557-83-5	1.27%	2.00%	-	-	-	-	-	-	-	-	-	-	-	N.A.
Ethyl Stearate	111-61-5	1.20%	1.76%	-	NIG	-	-	-	-	-	-	-	-	-	[48]
Stigmasterol	83-48-7	1.07%	1.25%	≤10%	-	-	-	-	-	-	-	-	-	-	[42]
Methyl cholesterol	4651-51-8	1.05%	1.25%	-	-	-	-	-	-	-	-	-	-	-	N.A.
Hexadecyl acetate	629-70-9	1.03%	1.08%	≤12.6%	-	-	-	-	-	>40 mL/kg	>5000	-	-	-	[49,50]
Ethyl linoleate	544-35-4	0.95%	2.00%	-	NIG	-	-	-	-	>2000 ^#^	>2000 ^#^	-	-	-	[51,52]
Benzyl linolenate	77509-02-5	0.79%	1.49%	-	-	-	-	-	-	-	-	-	-	-	N.A.
Benzyl hexadecanoate	41755-60-6	0.78%	1.38%	-	-	-	-	-	-	-	-	-	-	-	N.A.
Ethyl Palmitate	628-97-7	0.55%	1.27%	-	NIG	-	-	-	-	>2000 ^#^	>2000 ^#^	-	1000 ^#^	-	[53,54]

CIR = Cosmetic Ingredient Review, RIFM = Research Institute for Fragrance Materials, Inc., ECHA = European Chemical Agency, CAS = Chemical Abstracts Service, NOAEL = No Observed Adverse Effect Level, LD50 = Lethal Dose 50, NESIL = No Expected Sensitization Induction Level, Ref. = References, N.A. = Not Available, NG = Not Genotoxic, NIG = No Indication of Genotoxicity, NPT/A = not phototoxic/photoallergenic, # = read-across, † = sub-acute study, ‡ = sub-chronic study, * = chronic study, - = not available, ± = No ECHA Dermal Repeated Dose NOAEL was available for the compounds listed.

**Table 4:** Available information on commercial blue lotus products.

Sample	Oil Name	Description	Botanical Name
C1	Egyptian Sahasrana 100% Blue Lotus Oil Euphoria	100% Blue Lotus Oil Euphoria	NA
C2	Blue Lotus Oil	Therapeutic grade	NA
C3	Lotus Blue Oil	Pure essential oil, steam distilled	*Nymphaea caerulea*
C4	Blue Lotus Extra Strength	Euphoric mood + dream tonic and liquid tincture, glycerin, alcohol, filtered water	*Nymphaea c*. 200:1
C5	Blue Lotus Absolute	100% pure, natural, and undiluted EO	*Nymphaea caerulea*
C6	Blue Lotus EO	100% natural ingredients	NA
C7	Blue Lotus Oil	100% pure EO	NA
C8	Blue Lotus EO	NA	NA
C9	Blue Lotus absolute Oil	Organic • 100% PURE • Absolute	NA
C10	Blue Lotus essential Oil	NA	NA
C11	Blue Lotus essential Oil	NA	NA

NA = not applicable.

**Table 5:** Multiple reaction monitoring mode parameters (MRM).

Name	CAS #	Precursor (m/z)	Product 1 (m/z)	Product 2 (m/z)	Product 3 (m/z)	RT (min)	r^2^
Apomorphine	58117-94-5	309.05	268.20	237.15	191.1	1.47	0.9995
Nuciferine	475-83-2	296.00	265.10	250.10	235.15	2.87	0.9995

r^2^, equation and coefficient of determination.

## Data Availability

Data are contained within the article.
